# Spatial Patterns of Excitation at Tissue and Whole Organ Level Due to Early Afterdepolarizations

**DOI:** 10.3389/fphys.2017.00404

**Published:** 2017-06-22

**Authors:** Nele Vandersickel, Enid Van Nieuwenhuyse, Gunnar Seemann, Alexander V. Panfilov

**Affiliations:** ^1^Department of Physics and Astronomy, Ghent UniversityGhent, Belgium; ^2^Institute for Experimental Cardiovascular Medicine, University Heart Center Freiburg · Bad Krozingen, Medical Center, University of FreiburgFreiburg, Germany; ^3^Faculty of Medicine, University of FreiburgFreiburg, Germany

**Keywords:** cardiac arrhythmias, Early afterdeloparizations, spiral waves, cardiac modeling, Torsades de Pointes

## Abstract

Early after depolarizations (EAD) occur in many pathological conditions, such as congenital or acquired channelopathies, drug induced arrhythmias, and several other situations that are associated with increased arrhythmogenicity. In this paper we present an overview of the relevant computational studies on spatial EAD dynamics in 1D, 2D, and in 3D anatomical models and discuss the relation of EADs to cardiac arrhythmias. We also discuss unsolved problems and highlight new lines of research in this area.

## 1. Introduction

Early afterdepolarizations (EADs) are defined as a premature intrinsic depolarization of the transmembrane voltage before the completion of the repolarization (Huffaker et al., 2004; Roden and Viswanathan, 2005; Qu et al., 2013). EADs occur in many forms of genetic defects such as the long QT syndrome (Keating and Sanguinetti, 2001; Morita et al., 2008; Liu et al., 2012), under the action of pharmacological agents as result of cardiotoxicity (Volders et al., 2000; Sipido et al., 2007; Kannankeril et al., 2010), due to heart failure (Nuss et al., 1999; Li et al., 2002), and in several other conditions (Volders et al., 2000; Sipido et al., 2007). It is well-established that EADs are associated with increased arrhythmogenicity by producing focal beats which can initiate arrhythmia. In addition, EADs can augment the electrical heterogeneity in the heart which is also a well-known arrhythmogenic condition (Antzelevitch and Sicouri, 1994; Sato et al., 2009). Over the years, EADs have been widely studied in experimental (Volders et al., 2000) and in *in-silico* studies (Qu et al., 2013). Most of these experimental and theoretical studies have investigated the occurrence of EADs at the single cell level. However, arrhythmias occur at the tissue level and therefore it is very important to study EAD manifestations at the tissue and whole heart level.

Recently, several computational studies have been performed addressing important aspects of EAD dynamics in tissue and in whole heart under various conditions. However, these studies are still fragmented and do not provide a detailed picture of the possible spatial dynamics of cardiac tissue prone to EAD generation. The goal of this paper is to review the current status of these studies performed in 1D, 2D, and at the whole heart level. Additionally, we highlight unsolved questions and issues in this important area. Links to experimental studies will be made where possible.

The paper is organized as follows. In Section 2, we will discuss how EADs emerge in single cells, in Section 3 we will cover the different spatial patterns due to EADs in 1D, 2D, and the whole heart, in Section 4, we will discuss the importance of the rate dependency of EADs, and in Section 5, we overview the relation of EADs and Torsade de Pointes arrhythmias.

## 2. EADs at the single cell level

Before we consider spatial patterns due to EADs dynamics the occurrence of EADs in a single cell is briefly discussed. The onset of EADs at cellular level is a large area of theoretical and experimental research. In a very general sense, the onset of an EAD is the result of an imbalance of inward and outward currents during the repolarization phase of the action potential (AP). Therefore, they mainly occur as a result of an increased function of inwards currents (L-type Ca^2+^ current *I*_*CaL*_, or late sodium current *I*_*Na*_), or decreased function of the repolarizing potassium currents [mainly the rapid (*I*_*Kr*_) or slow (*I*_*Ks*_) delayed rectifier potassium current]. Such modifications are often referred to as the reduction of the repolarization reserve (RR) (Roden, 1998, 2008; Roden and Yang, 2005). In addition, EADs can occur indirectly via Ca^2+^ dynamics when a release of Ca^2+^ activates the Sodium-Calcium exchanger *I*_*NCX*_ (Volders et al., 2000). Recently, mechanisms including transient outward current (*I*_*to*_) were also identified (Zhao et al., 2012). Another important question, also studied at the cellular level, is the rate dependency of EADs. The two main classes include EADs which occur at low stimulation rate or EADs which occur at fast stimulation rate. Detailed information about cellular mechanisms of EAD can be found in other reviews (Volders et al., 1997; Weiss et al., 2010). Most of the studies reviewed in this paper consider EADs which occur as a result of increase in *I*_*CaL*_, as modification of *I*_*Na*_ or as a decrease of various components of *I*_*K*_.

**Figure 1:**
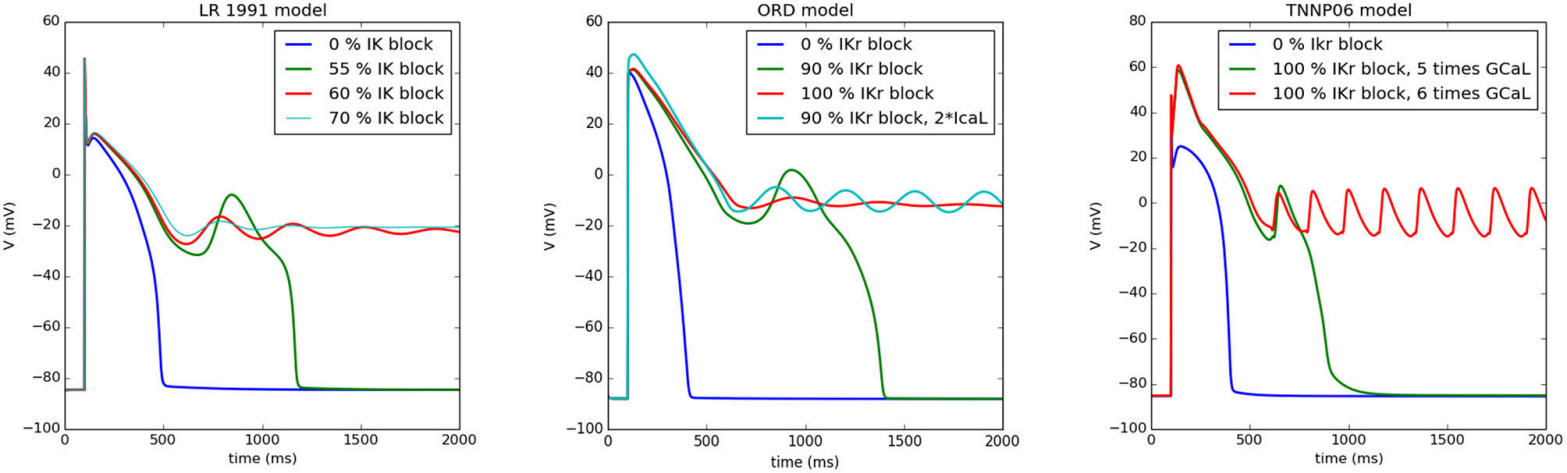
Different types of APs when reducing the RR as simulated by myokit based on CellML Clerx et al. (2016). In the left Figure, the Luo Rudy model (1991) was simulated: for normal parameters (0% *I*_*K*_ block), the standard AP was found, while for a 55% *I*_*K*_ block an EAD arises, for 60% block the AP behaves as a damped oscillator around a higher equilibrium, similarly for 70% block, but with a smaller amplitude. Similar behavior was found for the ORD model (middle panel) for 0% *I*_*Kr*_ block, for 90% block, for 100% block (damped oscillator), and for a 90% *I*_*Kr*_ block and 200% of the L-type Ca^2+^ current (oscillator). For the TP06 model (right panel), blocking *I*_*Kr*_ is not sufficient for the generation of EADs. Here, we have increased the L-type Ca^2+^ current. However, also similar behavior was found here, although the oscillator was not damped in this case. For 100% block and a 5 time increase of the L-type Ca^2+^ current we find an EAD, for a 6 times increase, the AP oscillates around a higher equilibrium state.

A typical situation is shown in Figure 1 for the Luo-Rudy model (LR91) (Luo and Rudy, 1991), the O‘Hara, Virag, Varro, Rudy model (ORD) (O'Hara et al., 2011), and the ten Tusscher, Panfilov (TP06) model (ten Tusscher and Panfilov, 2006). We first showed a normal AP which by gradual blocking of *I*_*K*_ gave rise to an increased APD. If we continuously reduced *I*_*K*_, we found an AP which showed one or multiple EADs and further RR reduction resulted in non-repolarizing APs. At this stage, the AP could either oscillate (damped or not-damped) around this new equilibrium, or it just maintained a constant potential, depending on the degree of *I*_*K*_ block (see Figure 1). From the viewpoint of a dynamical system, the onset of oscillations is associated with a Hopf bifurcation (Tran et al., 2009) and the formation of a non-repolarizing AP with other bifurcations changing the number of equilibria from 1 to 3 (Tran et al., 2009) (which most probably occurs via a saddle-node bifurcation). Interestingly, all three models show similar behavior, see Figure 1.

**Figure 2:**
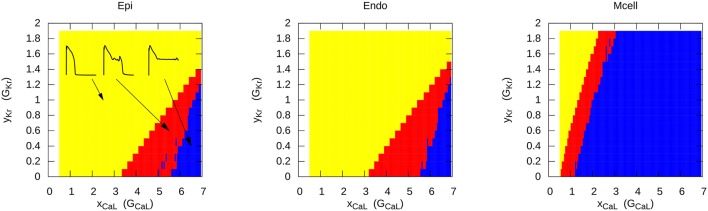
Different types of AP when reducing the repolarization reserve in the TP06 model for epicardial, endocardial and M-cells. Yellow represents the normal AP without EADs, red shows APs with one or multiple EADs, and in the blue region, the AP starts to oscillate around a higher equilibrium. The increase of the conductance of the L-type Ca^2+^ current (e.g., 2 times larger conductance) is presented on the x-axis, similar on the y-axis, the increase (> 1) or decrease (< 1) of the conductance of *I*_*Kr*_ is presented. Figure reproduced from Vandersickel et al. (2014) with permission.

The most representative way to show the conditions for EAD generation is to represent it in a two-parametric diagram. This was first done analytically in Tran et al. (2009) for a subsystem of the LR model. In more complex systems, it is necessary to find these regions numerically. This was performed for two models of human cardiac cells: For the TP06 (ten Tusscher and Panfilov, 2006) model and the ORD model (O'Hara et al., 2011). Figure 2 shows an exemplary 2-parametric diagram for EADs in the TP06 model for the endocardial, epicardial, and M-cell version (Vandersickel et al., 2014). The RR was reduced by decreasing *I*_*Kr*_ and increasing *I*_*CaL*_. Again, three different behavior of APs were found: the yellow region shows normal APs, the red region shows APs with one or more EADs, and the blue region shows APs which do no longer repolarize and oscillate around a less negative equilibrium. We see that the M-cells are much more prone to EAD generation.

A similar 2-parametric diagram was obtained for the endocardial cells in the ORD model (Zimik et al., 2015). As that of Figure 2 this dependency also had typical triangular shape in *G*_*Kr*_ *G*_*CaL*_ coordinates with similar corresponding dynamics. However, in the ORD model the single EAD regime occupied a smaller parametric range and in case of non-repolarizing activity the ORD model showed a small region with dumped oscillation regime. These quantitative differences are not significant as both models produce similar spatial excitation regimes (see results section in Zimik et al., 2015).

## 3. Spatial patterns due to EADs in 1D, 2D, and 3D

As EAD action potentials substantially differ from normal APs, it is important to understand how such abnormal shapes will affect the spatial patterns of excitation. In this section, we will describe all known spatial patterns which occur in 1D, 2D systems, and in anatomical models of ventricles of the heart.

### 3.1. 1D patterns

**Figure 3:**
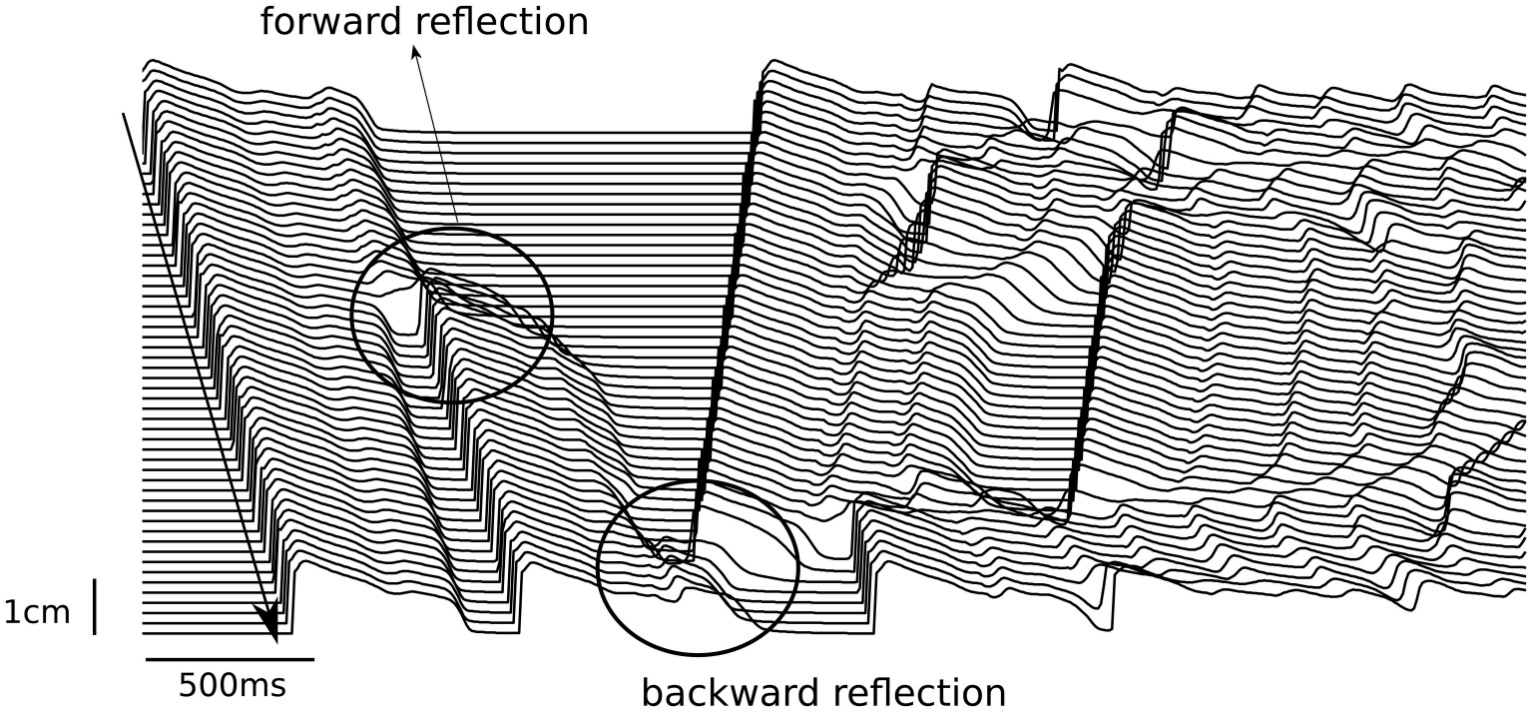
Forward and backward reflection due to EADs. The pseudo 1D tissue had a length of 10 cm (y-axis). A single stimulus was given at the top side of the tissue (top left of the figure), and propagated to the bottom. At *t* = 0.7 s, an EAD developed into a triggered beat (forward reflection) and propagated to the bottom. At *t* = 1.42 s, another EAD developed into a triggered beat, but propagated back to the top (backward reflection).

If we consider an action potential with a single EAD response and study its propagation in 1D, we may expect that during wave propagation this EAD response will either be kept, will grow or EAD shape will disappear. If it grows, it may form a new wave, which will either propagate forward, along the first wave, or will be reflected backward. All these regimes can easily be obtained in any model of cardiac tissue. Figure 3 shows them in the TP06 (ten Tusscher and Panfilov, 2006) model whereby the RR was gradually reduced in a pseudo 1D tissue of 10 cm length. The tissue was paced from the left only once, and the wave propagation was observed. The RR was reduced by increasing the conductance of the inward current *I*_*CaL*_ and by blocking the outward current *I*_*Kr*_, see also (Vandersickel et al., 2014). At a four-fold increase of *I*_*CaL*_, a single stimulus triggered forward reflection as well as backward reflection (Figure 3). These patterns of forward and backward reflections continued and resulted in sustained electrical activity. Moreover, for different parameter values more complex patterns of simultaneous multiple forward and backward reflections were also observed.

Historically, the formation of additional excitations was first studied in relation with the reflection phenomenon (Moe et al., 1982). Modeling of reflections (see e.g., Cabo and Barr, 1992; Aslanidi and Mornev, 1996; Mornev et al., 1996) revealed that in heterogeneous and even in homogeneous tissue the propagating wave can be reflected from the boundary, or two colliding waves can pass through each other (Aslanidi and Mornev, 1999). It was also shown that this phenomenon is closely related to the Hopf bifurcation in the system (Aslanidi and Mornev, 1999) and has a lot of similarities to the processes which occur in EAD sensitive media. However, in most of the cases the additional pulse in these systems occurred at a late stage of the action potential, namely phase 4, normally 2 s after the propagating pulse collided with the boundary, and thus it must be classified as delayed afterdepolarizations (DADs) related phenomenon.

In relation to EADs, forward reflection was first presented in Huffaker et al. (2004), where the rotation of wave in a 1D ring was studied. The authors found that during propagation of a wave an EAD can form an additional wave propagating in the same direction as the primary wave. As a result there were two waves rotating in the same direction. However, in some situations, the ring of a given size could not sustain two waves due to spatial limitation. As a result, either both waves disappeared, or only one wave (the secondary EAD wave) remained, while the primary wave annihilated by collision with the waveback of the secondary wave. EADs were generated using the Chudin et al. model (Chudin et al., 1999) (based on LR91), by decreasing *I*_*Kr*_ (long QT Syndrome 1–LQT1) and reducing *I*_*Ks*_ (LQT2) additional to a modest increase of *I*_*CaL*_ and *I*_*pCa*_. The EADs generated were fast rate dependent: they occurred for smaller ring lengths and the mechanism of EADs was due to Ca^2+^ overloading.

Later, also backward reflections were found in the same setup but with different alterations (Huffaker et al., 2007) (namely by modification of dynamics of the non-selective calcium current). Moreover, also more complex behavior was observed, when more than one wave was generated simultaneously in forward direction and backward direction.

Forward or backward reflections were also observed in heterogeneous 1D systems where a small region with predescribed EAD dynamics was present inside the normal tissue (Scarle and Clayton, 2009). Depending on the size of the EAD region, the EAD period, and the EAD amplitude this EAD region generated new waves which propagated in the opposite direction and in most of the cases were also followed by waves in the same direction of the primary wave. In Maruyama et al. (2011) another 1D system with a step-wise heterogeneity was studied, where cells with a short AP were coupled with cells with prolonged AP which had small AP phase-2 EADs. It was found that AP phase-3 EADs emerged at the boundary zone between these two types of cells. Upon further reduction of the RR, cells in the region with longer AP failed to repolarize resulting in triggered activity when the waves were periodically emitted backwards from the boundary region.

In general the reflection phenomenon is not necessarily related to EAD formation. Experimentally, the reflection was demonstrated on various preparations of Purkinje fibers, which involved a zone of slow conduction and the reflected wave was generated due to a delay in the transmission of the pulse through this zone. This delay permitted the recovery of the tissue and retrograde pulse propagation (Antzelevitch, 1983; Antzelevitch and Burashnikov, 2011). In this review paper, we demonstrate that reflection naturally occurs in the presence of EADs and underlies the spatial excitation dynamics.

In conclusion, the main 1D manifestations of EADs are forward and backwards reflections. However, it remains to be studied, what the necessary conditions for each of these regimes are and how they are related to the parameters of the model and the RR.

### 3.2. 2D spatial patterns

In this section, we present on overview of possible 2D spatial patterns due to EADs.

#### 3.2.1. 2D spatial patterns in homogeneous media

**Figure 4:**
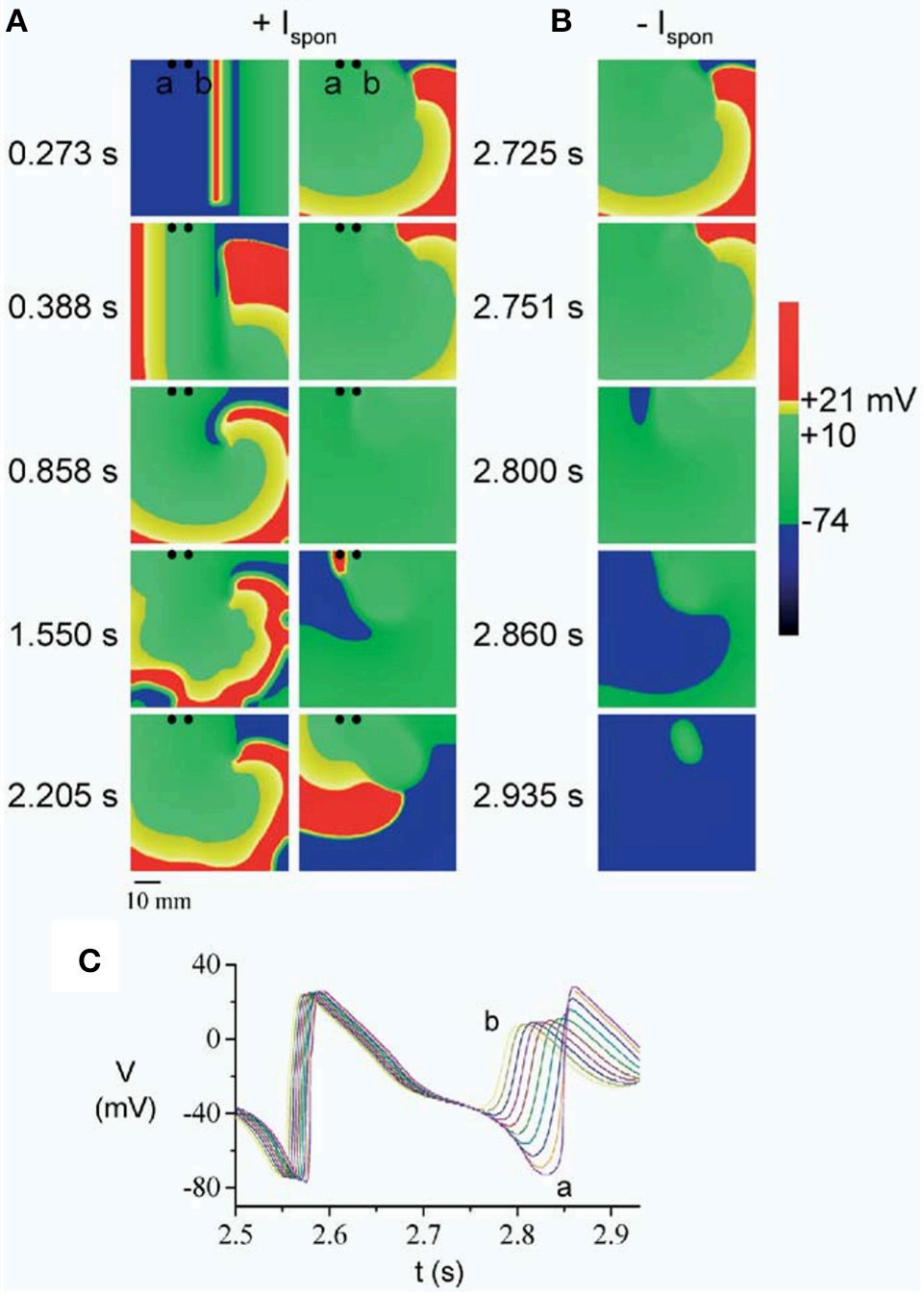
A spiral wave initiated by S1S2 stimulation, started to meander due to reduction of the repolarization reserve. Upon collision with the boundary, the spiral wave was regenerated **(A)** by an EAD caused by spontaneous Ca^2+^ release (at *t* = 2.860 s), which is mediated through the current *I*_*spon*_. In **(B)** the spontaneous release was removed (*I*_*spon*_ = 0) and no regeneration was observed. The times labels in between **(A,B)** is for both the left and the right side. The corresponding EADs are shown in **(C)**. Figure reproduced from Huffaker et al. (2004) with permission.

In a first series of papers by Kogan et al. the effect of EADs on spiral waves in 2D was investigated. In Huffaker et al. (2004), a spiral wave was generated by the S1S2 protocol in the LR91 model with modification of Chudin et al. (1999) and *I*_*Kr*_ or *I*_*Ks*_ were reduced to reproduce the effect of the LQT1 or LQT2 mutations. These modifications increased the meandering of the spiral wave. As a result of the meandering, the spiral wave could eventually approach the boundary of the tissue and collide with it, as shown in Figure 4. Upon crossing the boundary, the spiral wave did not disappear as occurs in normal cardiac tissue, however it was regenerated in the same direction due to triggered activity caused by EADs (Similar to the 1D wave reflection from the boundary discussed in the previous section). It should be noted that for these simulations, EADs were fast rate dependent, and generated due to spontaneous release of Ca^2+^ from the SR.

Similar results were found in Kogan et al. (2003), where the same model was studied, but another mechanism of EAD initiation was investigated, namely an increased sensitivity of the non-selective Ca-current *I*_*ns*(*Ca*)_ to the intracellular Calcium concentration ([Ca^2+^]_*i*_). It was found that after spiral wave initiation, accumulation of Ca^2+^ in the SR occurred, followed by spontaneous releases of calcium which increases the AP duration (APD). This resulted in the annihilation of the spiral wave via front-tail interactions. Important to note, the manuscript does not clearly state whether this prolongation was accompanied by the formation of EADs. However, EADs did emerge after the wave disappeared, due to spontaneous releases of Ca^2+^ from the SR and regenerated another spiral. This new spiral could have the same or the opposite direction of rotation as the original spiral wave. This process of spiral formation and disappearance continued a few times before final disappearance of the spiral.

Another simulation in the same model which investigated the effect of EADs on spiral waves was performed in Huffaker et al. (2007). There, the spiral wave was stationary until EADs occurred. EADs first appeared in the arm of the spiral wave near the core, and as the EADs prolong refractoriness, the spiral tip was displaced causing meandering. Such meandering could result in self-termination of the spiral wave by its collision with tissue borders. However, after collision with the boundary the spiral wave could be reinitiated in some cases (as in paper Kogan et al., 2003 discussed above). Summarizing, it might mean that there are two types of meandering of the spiral wave, (1) due to non-uniform prolongation of the APD (Kogan et al., 2003; Huffaker et al., 2004), and (2) due to EAD formation in the core (Huffaker et al., 2007). In Vandersickel et al. (2015), also such regeneration of spirals was observed where the defibrillation of spiral wave patterns were studied which were maintained by EADs. It was found that in many cases, in tissue which is prone to EADs, spiral waves can be removed but are regenerated. This resulted in an increase of the threshold of defibrillation.

**Figure 5:**
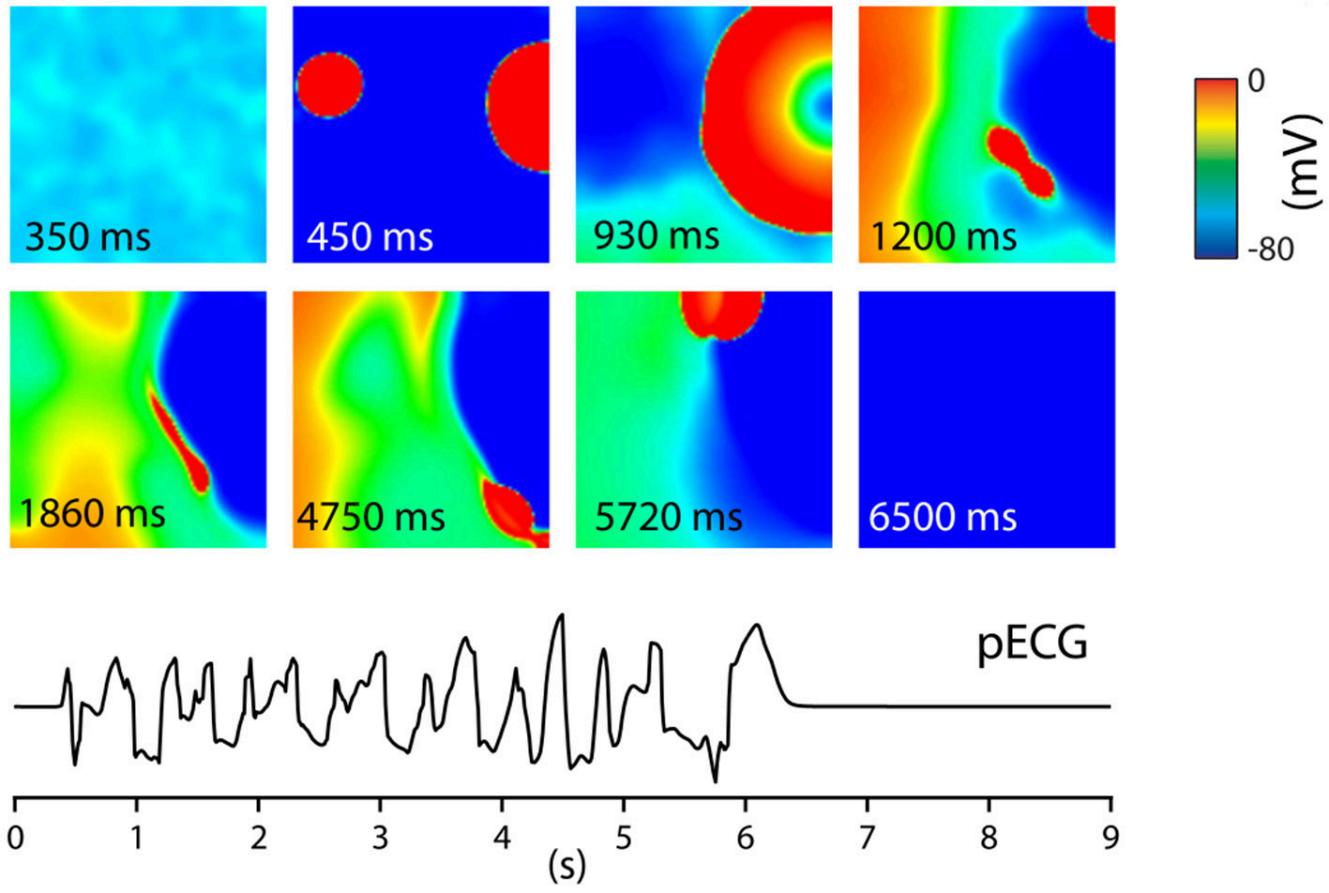
Tissue of 600 by 600 cells. The entire tissue was stimulated at *t* = 0 s. After this stimulus, at *t* = 450 ms, EADs generated focal beats. These varied dynamically in time and space giving, until at *t* = 6,500 no new EAD activity was measured. This gave rise to a PVT like pseudo ECG. Figure reproduced from de Lange et al. (2012) with permission.

Other possible spatial EAD patterns are organized by purely focal activity, or mixed focal and reentry patterns. These regimes were studied in a series of papers (Sato et al., 2009; Chang et al., 2012, 2013; de Lange et al., 2012; Kim et al., 2015), using a rabbit cell model (Mahajan et al., 2008) which was modified to show EADs. For that, the dynamics of *I*_*CaL*_ were modified by making its activation and inactivation kinetics steeper and by increasing the maximum conductance of *I*_*CaL*_. In addition, the maximum conductance of *I*_*Ks*_ was reduced. This model showed slow rate dependent EADs. In the first paper in this series (Sato et al., 2009), 2D and 3D simulations were performed. It was shown that EADs occurred irregularly due to an underlying chaotic behavior in the dynamical system. Although, the system was homogeneous, partial regional synchronization of chaotic EADs generated localized focal excitations and could generate purely focal or mixed focal-reentrant arrhythmias in homogeneous tissue. The main mechanism was the following: EAD first formed a focal beat which in 2D and 3D originated at a certain location. The wave formed by this focal beat generated another focal beat at another location and so forth. This led to spontaneous formation of multiple foci that vary dynamically in time and space (see Figure 5 for an example). In this Figure, one can see how two focal beats arose (450 ms) after the tissue was stimulated, and continued to arise in the tissue until no more activity was observed after 6,500 ms. The pseudo ECG had characteristics of polymorphic ventricular tachycardia (PVT). This behavior was confirmed by experimental research in a rabbit model of LQT1 (Kim et al., 2015). Similar computational results were obtained in de Lange et al. (2012). In some simulations the focal activity led to multiple spiral waves, while in others, the triggered activity due to multiple focal sites stopped spontaneously. An interesting property of the waves was that they showed bi-excitability, meaning that some of the waves were I_*Na*_-mediated (fast) wavefronts, while others were I_*CaL*_-mediated (slow) wavefronts (Chang et al., 2012, 2013).

**Figure 6:**
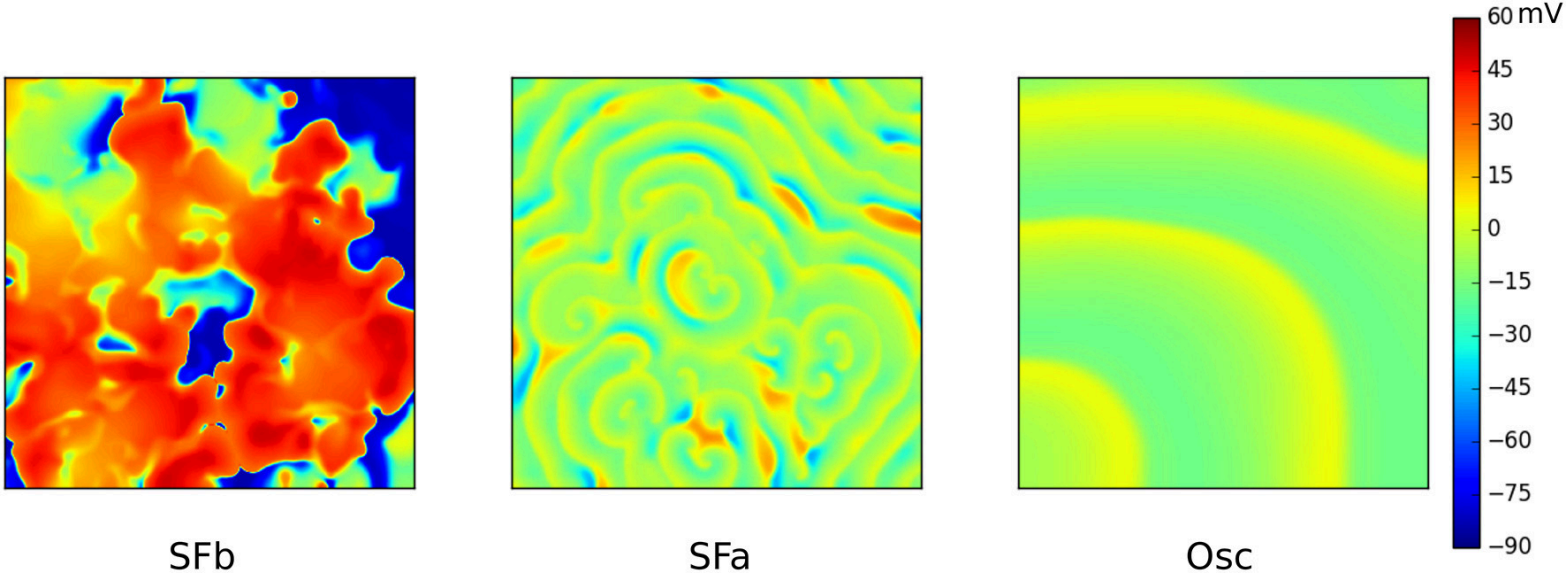
The three different types of waves patterns due to EADs in the TP06 model. SFb **(left)** consist of short living spirals where *I*_*Na*_-mediated and *I*_*CaL*_-mediated waves coexist. The conductance of *I*_*CaL*_ was increased 3.5 times, *I*_*Kr*_ was 10% blocked. SFa **(middle)** only consists out of *I*_*CaL*_-mediated waves. Conductance of *I*_*CaL*_ was increased to 4.5 times, *I*_*Kr*_ was 70% blocked. The Oscillatory type **(right)** consisted solely out of phase waves. Same parameters as for SFa, however *I*_*Kr*_ was now fully blocked. Figure composed from the data generated in Vandersickel et al. (2014).

An effective way of studying possible wave patterns due to EADs is by generating a full parametric study whereby RR is gradually reduced. In Vandersickel et al. (2014) and Zimik et al. (2015) this was done for two different human ventricular models, the TP06 model (ten Tusscher et al., 2004) and the ORD (O'Hara et al., 2011) model. Although, the two models displayed different properties, very similar patterns were found. By gradually decreasing the RR (by decreasing *I*_*Kr*_ and increasing *I*_*CaL*_), three different types of such arrhythmias were characterized. First, a fibrillatory pattern was found, namely, spiral fibrillation type b (SFb), see Figure 6, which is also characterized by the coexistence of *I*_*Na*_-mediated waves with *I*_*CaL*_-mediated waves (Chang et al., 2012). Only short-living rotors were observed very often in this pattern (which do not even make one rotation) and this pattern had a very chaotic nature. It had some resemblance with the mixed focal-reentry pattern seen in Sato et al. (2009), although in the latter pattern, more long lived rotors were observed. Reducing the RR further, a second fibrillatory pattern was found, called spiral fibrillation type a (SFa), see Figure 6. There, spiral waves were clearly observed; however, they are purely maintained by *I*_*CaL*_-mediated waves, similar as in de Lange et al. (2012), whereas almost no *I*_*Na*_ was present anymore. Finally, reducing the RR to such an extent that the single cells in the 2D tissue no longer repolarize to their resting state, patterns of an oscillatory type (Osc) that consists out of phase waves were found (see Figure 6). Important to note is that the initial conditions did not have an influence on the final pattern. However, there were quantitative differences in the wave patterns of each wave type. In Zimik et al. (2015), very similar simulations were performed for the ORD model of human ventricular cells. It was shown that the same three types of waves patterns (SFa, SFb, and Osc) occur. However, there were some differences. The SFb type in the ORD model showed short-lived spirals, but in the TP06 model the spirals were even shorter lived, as they do not even make one rotation. For the SFa type, the TP06 model supported more *I*_*CaL*_-mediated spirals than those in the ORD model. For the oscillatory type, the TP06 model exhibited more phase-wave patterns than the ORD model. However, we considered these differences as not significant as they do not qualitatively change the observed types of spatial wave dynamics.

#### 3.2.2. 2D spatial patterns in heterogeneous tissue

It is well-known that the heart is a heterogeneous medium. There exist many types of heterogeneities, such as the transmural heterogeneity (Liu and Antzelevitch, 1995; Zygmunt et al., 2000; Burton and Cobbe, 2001), a base to apex heterogeneity (Burton and Cobbe, 2001), etc. The transmural heterogeneity is related to the difference in properties of endocardial, epicardial, and M-cells, and it was shown that the M-cells are more prone to emergence of EADs than other cells types (Sicouri and Antzelevitch, 1991, 1993) (see also Figure 2). Therefore, the onset of EADs in the presence of heterogeneities was studied in a number of papers (Fenton et al., 2002; Scarle and Clayton, 2009; Xie et al., 2010; Auerbach et al., 2011; Vandersickel et al., 2016). Most of these studies considered a case when a single localized heterogeneity prone to EADs was added to the normal cardiac tissue. In Scarle and Clayton (2009), these localized EADs were not generated from the solution of an ionic model of cardiac cells, but they were reproduced by a pre-assigned function of a transmembrane voltage mimicking the time course of AP with various types of EADs. Such phenomenological approach allowed the authors to change properties of EADs in a very controlled way. They could change the number of EADs, their amplitude and their period. Outside the EAD region the authors used a 4-variable ionic model for canine cardiac tissue (Fenton et al., 2002). It was found that the EAD region could generate focal beats. The authors argued that varying the spatial size of the EAD region did not affect the outcome. Although, this conclusion is logical for the heterogeneity of a large size, it was widely assumed that there is a minimal size of an EAD region which is capable to generate focal beats. In Scarle and Clayton (2009), the minimal investigated size in 2D was 314 *mm*^2^ and such EAD region was capable to produce focal activity. Taking a cell size of 100μ*m* by 10μ*m* we find that it results in ≈100,000 cells. Increased EAD amplitude and increased time of EADs being present in the tissue resulted in a larger amount of propagating extra beats coming from the heterogeneity. It was also found that in some cases an extra beat was able to re-enter the heterogeneity and create stable reentry. A longer total duration of the EADs in the heterogeneity and shorter periods of a single EAD favored this reentry-type.

One of the most important questions regarding 2D dynamics is how EADs can initiate focal or reentrant activity in cardiac tissue. Although, formation of focal activity by EAD cells seems straightforward, one important issue here is that even if a single cell produces an additional AP, in order to excite the surrounding tissue it needs to overcome the electrotonic load from the neighboring cells. A detailed analysis of this problem was performed in Xie et al. (2010) where it was shown that a certain number of synchronous EADs are necessary to produce a focal beat. To obtain EADs, the parameters of a rabbit cell model (Mahajan et al., 2008) were adapted similarly as in Xie et al. (2010). In addition, some changes to the transient potassium outward current *I*_*to*_ were made. It was found that in 1D a focal beat can be produced by 70 cells, in 2D by about 7,000 cells, and in 3D by 700,000 cells. Hence, the size of the EAD region does play a critical role in the ability to produce focal beats and varies greatly depending on the number of dimension. Therefore, it is logical that a structural heterogeneity can increase the propensity to EADs. For example a thin strand of cells (isthmus) which connects two wide regions of tissue represents itself a 1D structure in 3D environment and can generate EAD focal beats in tissue more easily (Auerbach et al., 2011).

The next important issue is the generation of spiral waves. Here, several scenarios are possible. Due to the formation of EADs, the duration of an AP can be substantially increased in a certain part of the tissue. Therefore, it can create large heterogeneities in cardiac tissue. The waves can break at these heterogeneities and form spiral waves. For example in Yang et al. (2010) it was shown that short-long-short stimulation induces a reentry source due to the break of the wave in the M-cell region prone to EADs. Another classical mechanism of reentry initiation is the S1S2 stimulation protocol. Here the spiral wave is generated by an additional stimulus applied the end of another propagating wave (Tusscher and Panfilov, 2003). EADs can generate such an S2 stimulus by backward reflection and therefore create spiral waves in the tissue. Examples of this dynamics can be seen in many modeling studies, for example in Vandersickel et al. (2014), de Lange et al. (2012), Scarle and Clayton (2009), and Qu (2011).

In Vandersickel et al. (2016) the effect of several EAD-prone heterogeneities was investigated on the 2D excitation patterns. These heterogeneities had sizes based on experimental measurements (Glukhov et al., 2010) and reduced the RR due to increasing *I*_*CaL*_ and reducing *I*_*Kr*_ in comparison with the surrounding tissue. Firstly, heterogeneities were considered with a large difference in RR in comparison to the surrounding tissue (*I*_*CaL*_ inside the heterogeneity increased to 2.15 times, *I*_*Ks*_ reduced by 25%). Then upon reducing the RR in whole tissue simultaneously, these heterogeneities acted as localized sources of focal activity. This is in contrast with moving sources observed in Sato et al. (2009), de Lange et al. (2012), and Kim et al. (2015). Although, heterogeneities had different sizes, there was no domination of larger heterogeneity and focal activity was originating from various heterogeneities from various places and showed very complex dynamics with an ECG pattern of a PVT. In a second set of simulations, heterogeneities with smaller difference in RR in comparison to the surrounding tissue were considered. In that case, sustained focal activity was not observed, but instead formation of spiral waves was found. These spiral waves anchored at the heterogeneities and were rotating around them in a complex way which resulted in an ECG signal which resembles Torsade de Pointes (TdP).

### 3.3. Whole heart spatial patterns

#### 3.3.1. Whole heart spatial patterns in homogeneous media

Only very few studies have investigated spatial patterns due to EADs in the whole heart. In Sato et al. (2009), spatial EAD activity was studied in an anatomical model of the rabbit heart. Similar to 2D simulations it was found that spatial patterns were mainly organized by focal activity. This gave rise to an ECG with the characteristics of PVT, but the ECG did not show a smooth twisting of the axis, as is often observed during TdP.

#### 3.3.2. Whole heart spatial patterns in heterogeneous media

**Figure 7:**
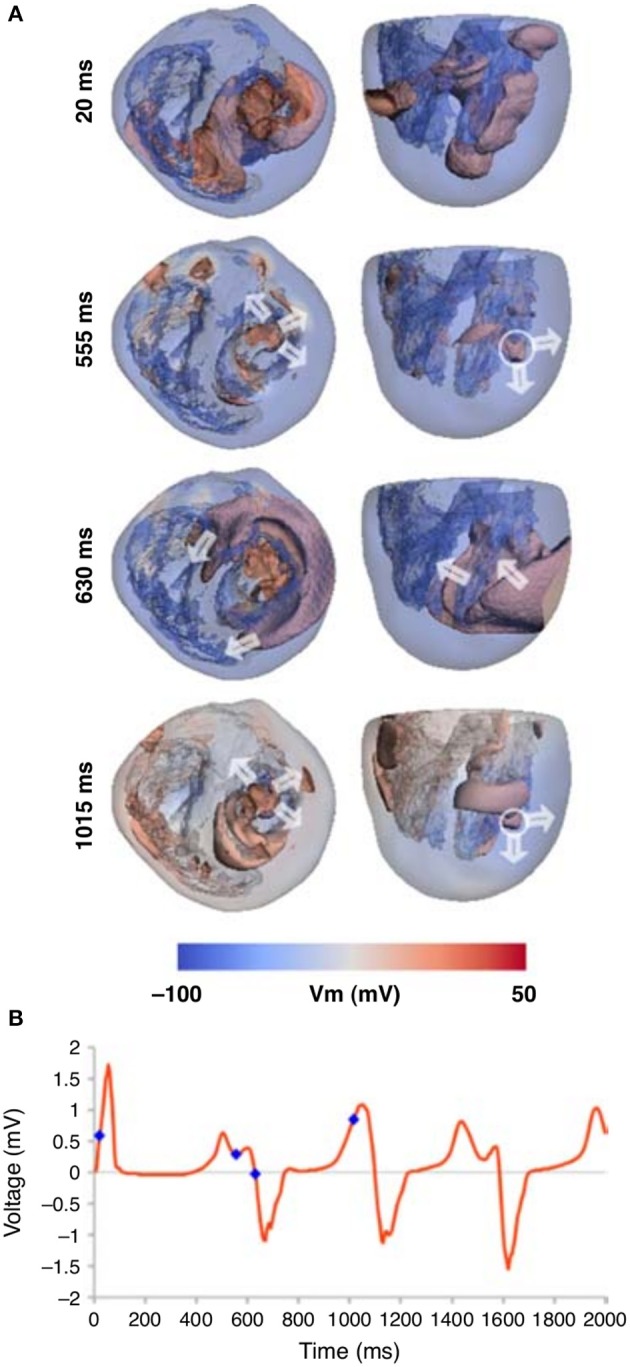
Whole heart computation with islands of M-cells, which show spiral wave generation due to electrically unstable M-cell islands **(A)**. **(B)** The corresponding ECG traces are shown for the Lead 1, with the time points marked that correspond to the image times in **(A)**. Figure reproduced from Richards et al. (2013) with permission.

General mechanisms of the onset of arrhythmias in heterogeneous models of the human heart were studies in several papers (Keldermann et al., 2008, 2009). In relation to the EAD dynamics, a heterogeneous human heart model was studied in Richards et al. (2013), where the islands of M-cells were added based on sizes of Glukhov et al. (2010). The authors modeled conditions of the LQT3 syndrome by enhancing the late Na^+^ current. They observed that in such conditions moving reentrant waves were initiated by the M-cell regions, see Figure 7. It would be interesting to prolong the simulation time, to see if the ECG shows similarities with a TdP-like ECG.

Also, whole heart simulations were done in Vandersickel et al. (2016), where heterogeneities of sizes based on Glukhov et al. (2010) were added, which had a reduction of RR in comparison with the surrounding tissue. As in the 2D studies reviewed above, two cases were considered: smaller or larger difference in RR in comparison to the surrounding tissue. As in the 2D case, for a “large” heterogeneity, the production of focal beats was observed, which resulted in very complex dynamics giving rise to PVT morphology in the ECG. Secondly, if the heterogeneity was “smaller,” the heterogeneities generated focal beats which degenerated into reentry due to conduction block formed by island of EADs in the surrounding tissue. This reentry had a very complex shape and rotation dynamics, also here the ECG resembled the ECG of TdP.

## 4. Rate dependency

So far, not much attention has been given to the rate dependency of EADs in computational studies. At least two different types of EADs exist, EADs which are fast and EAD which are slow rate dependent (Volders et al., 2000). To clarify, EADs which are slow rate dependent only appear at slow rates, and vanish at faster rates, while the opposite happens for fast rate dependent EADs. Typical examples of fast rate dependent EADs are EADs generated in canine myocytes by isoproterenol, which enhances *I*_*CaL*_. They are generated by spontaneous release of calcium from the SR (Volders et al., 1997). A typical example of slow rate dependent EADs are EADs generated by the *I*_*Kr*_ blocker almokalant. They are generated due to reactivation of *I*_*CaL*_ and occur when the APD becomes longer at slower rates (Volders et al., 1997). In computational studies (Kogan et al., 2003; Huffaker et al., 2004, 2007) the EADs were fast rate dependent. Although, the authors did not study onset of EADs at different stimulation periods, they investigated the rotation of the wave in a ring of different radius and found that EAD occur only if the period of the rotation is short enough. This is in contrast with later studies, were EADs were slow rate dependent (Fenton et al., 2002; Chang et al., 2012, 2013; de Lange et al., 2012; Kim et al., 2015). Interestingly enough in studies (Kogan et al., 2003; Huffaker et al., 2004, 2007) mainly spiral waves were induced, while in study (de Lange et al., 2012; Kim et al., 2015) patterns were mainly of focal activity. However, it was not specifically studied if different rate dependency contributed to different spatial patterns. In Vandersickel et al. (2014) and Zimik et al. (2015), the rate dependency did not have a substantial effect. In Vandersickel et al. (2014), the EADs in TP06 model were fast rate dependent, while in Zimik et al. (2015) EADs in ORD model were slow rate dependent. However, both models showed similar spatial dynamics. In general, the effect of the rate dependency on the spatial pattern organization is not yet fully investigated and is definitely an interesting future area of research.

## 5. EADs and TdP

**Figure 8:**
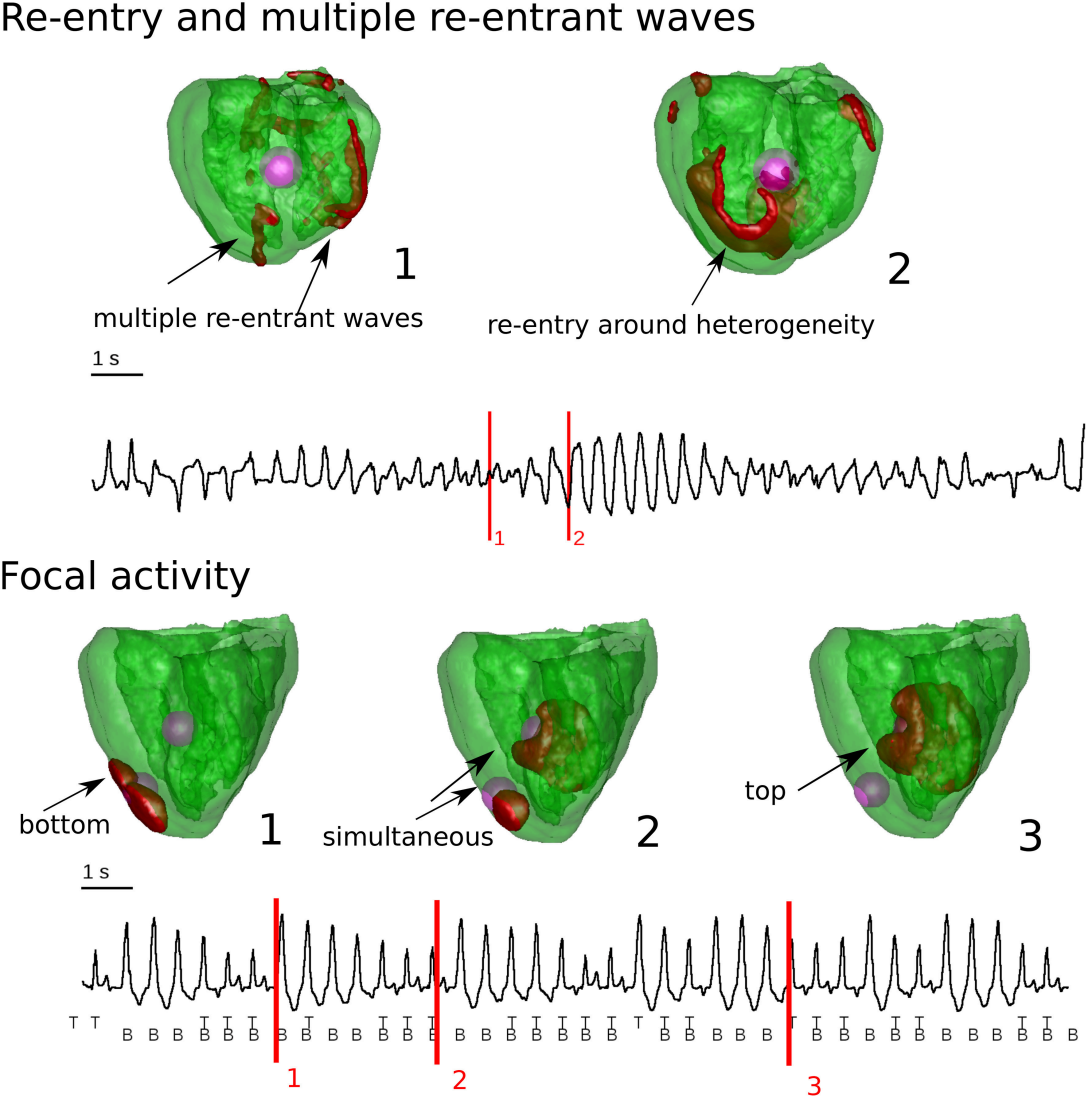
Whole heart computations with heterogeneities and reduced repolarization reserve in comparison to the surrounding tissue. In the top Figure, chaotic waves and reentry were responsible for the typical varying amplitude of the ECG. In case there was reentry, is was anchored around the heterogeneity. In the bottom Figure, the heterogeneities had a complex interaction which generated purely focal activity from the heterogeneities. This also gave rise to varying amplitude in the ECG. Figure composed from the data generated in Vandersickel et al. (2016).

One of the most important questions regarding spatial EAD dynamics is its relation to TdP. A TdP is a prevalent subtype of a polymorphic arrhythmia which can be lethal if not treated and which often occurs in the same conditions under which EADs are generated. Here we do not aim to present a comprehensive review on TdP mechanisms and dynamics, but we want to make a connection of the discussed EAD related patterns to this problem. In a very general way, TdP is characterized by a typical “twisting of the points” on the ECG, thus it must be related to some non-stationary excitation patterns. In experimental studies, both focal activity (Asano et al., 1997; El-Sherif et al., 1997; Derakhchan et al., 1998; Akar et al., 2002; Kozhevnikov et al., 2002; Boulaksil et al., 2011) as well as spiral waves (Murakawa et al., 1997; Senges et al., 2000; Schmitt et al., 2001; Choi et al., 2002; Schreiner et al., 2004) were found to be responsible for the underlying dynamics of TdP. Also in theoretical studies, both hypotheses were investigated. First it was demonstrated for re-entrant activity that heterogeneity-induced drift of a spiral wave (Rudenko and Panfilov, 1983; Panfilov and Vasiev, 1991) can induce a PVT (Gray et al., 1995) or a TdP-like ECG (Abildskov and Lux, 1991, 2000). However, in the latter studies, this 2D pseudo ECG does not have the typical amplitude variations often seen in the ECG of a TdP, and cannot be considered as solid evidence. In Vandersickel et al. (2016), it was shown that a spiral wave which changed rotation, also produced a TdP-like ECG in 2D and in an anatomical human heart model (see Figure 8), whereby we also found the typical amplitude variation in the ECG. In this case, the change in spiral wave rotation was due to properties of the medium which showed EADs, as well as due to heterogeneities added to the medium to which the spiral wave was anchored.

Recently, a very convincing TdP was presented in the Supplementary Material of Okada et al. (2015). In this study, the ORD model was used and different drugs were simulated to test their arrhythmogenic risk. Although, the authors did not study EADs in particular, in the specific example presented, *I*_*Kr*_ was blocked which caused EADs in the ORD model (Zimik et al., 2015). Although, not studied in detail, in the Supplementary Movie, one finds multiple spiral waves causing this TdP like signal.

For focal activity, not many *in-silico* studies have addressed the relation to the typical TdP-like ECG. In de Lange et al. (2012), where focal beats dynamically originated from different locations, a PVT-like ECG was shown, and similar in Vandersickel et al. (2016), where focal beats alternating in a complex way from fixed heterogeneities also gave rise to PVT like ECGs (see Figure 8).

From the above, it should be clear that the relation between TdP and the underlying mechanism is still not fully understood. Especially, more studies are necessary on the relation of the underlying mechanism of TdP in anatomically accurate models with accurate calculation of the 12 lead ECG. It will allow the researcher to relate more closely specific spatial wave excitation patterns to the clinically observed phenomena. It well may be that different mechanisms give rise to different morphologies of the ECGs, which are now all classified as TdP. Currently there is even no clear definition on how exactly a TdP in the ECG looks like and if it can be mechanisms specific.

## 6. Conclusions

The reviewed articles showed that when EADs are generated in single cells and are coupled to form tissue, complex spatial dynamics can arise. Forward and backwards reflection of the waves were observed in 1D. Moreover, very complex mixtures of forward and backward reflection are possible. These reflections most probably generate spiral waves in 2D tissue. However, in 2D these processes are not studied and it remains to be seen what the general mechanisms of generation of spirals due to EADs are. Overall the following spatial patterns were observed in 2D: (1) spiral waves meandering due to EAD activity in the core of the spiral wave, (2) spatially moving focal sources, (3) mixed (*I*_*CaL*_-mediated and Na-mediated) focal and reentry patterns (4) solely -mediated waves, and (5) phase waves.

In the whole heart only very few simulations have been performed. In homogeneous hearts the following was found: (1) purely focal activity in a rabbit model, (2) spiral wave activity initiated and altered by M-cell regions in a human model. We have also shown examples of (3) a chaotic pattern due to EADs and short living rotors (SFb), (4) purely *I*_*CaL*_-mediated waves, and (5) phase waves.

There exists a whole range of possible effects to be studied. An important question is role of the specialized ventricular conduction system of the heart in the onset of cardiac arrhythmias via triggered (EAD) dynamics (Haissaguerre et al., 2016). There are several factors which make the conduction system more prone to EADs. One of them is the duration of the action potential. First of all, it is generally assumed that the APD in the Purkinje cells which organize the conduction system is longer than that of working myocardium (Gaborit et al., 2007). In addition, the specific quasi one dimensional structure of the distal Purkinje network makes it possible for EADs to trigger ectopic beats (Xie et al., 2010). There are also substantial differences in Ca dynamics in the Purkinje cells which make them more prone to DAD activity (Haissaguerre et al., 2016). Clinically, triggered activity in the Purkinje network is an important factor for the onset of various arrhythmias in patients with or without structural heart disease (Haïssaguerre et al., 2008; Haissaguerre et al., 2016). However, the progression of EAD events to formation of arrhythmias is poorly understood, and mathematical modeling, especially including anatomically accurate modeling, can be an important tool to study these phenomena.

One of the most important questions regarding spatial EAD patterns is to establish a link between the EAD dynamics and TdP. It is extremely important to find the underlying mechanism which accounts for initiation and perpetuation of this arrhythmia for finding better treatments of this arrhythmia.

## Author contributions

AP, NV, and GS written the main text if the manuscript. EV did simulations supervised by NV and partially using software provided by GS.

### Conflict of interest statement

The authors declare that the research was conducted in the absence of any commercial or financial relationships that could be construed as a potential conflict of interest.
